# Therapeutic Potential of Chick Early Amniotic Fluid in Mitigating Ionizing-Radiation-Induced Damage

**DOI:** 10.3390/biomedicines13051253

**Published:** 2025-05-21

**Authors:** Ke Zhang, Hai Yang, Yueyue Wu, Yining Zhao, Wenxu Xin, Deshen Han, Ning Sun, Chao Ye

**Affiliations:** 1 Department of Basic Medicine, Wuxi School of Medicine, Jiangnan University, Wuxi 214122, China; 7232808009@stu.jiangnan.edu.cn (K.Z.); sysuyanghai@163.com (H.Y.); 1282220107@stu.jiangnan.edu.cn (Y.W.); 6232803013@stu.jiangnan.edu.cn (Y.Z.); 6222803020@stu.jiangnan.edu.cn (W.X.); 1282220417@stu.jiangnan.edu.cn (D.H.); 2 MOE Medical Basic Research Innovation Center for Gut Microbiota and Chronic Diseases, Wuxi School of Medicine, Jiangnan University, Wuxi 214122, China; 3 Department of Cardiovascular Medicine, The Affiliated Wuxi Children’s Hospital of Jiangnan University, Wuxi 214023, China

**Keywords:** ionizing-radiation-induced damage, chick early amniotic fluid, inflammation, oxidative stress, DNA damage and repair

## Abstract

**Background:** Clinical data indicate that at least half of patients with malignancies receive radiotherapy. While radiotherapy effectively kills tumor cells, it is also associated with significant ionizing radiation (IR) damage. Moreover, the increasing emissions of nuclear pollutants raise concerns about the potential exposure of more individuals to the risks associated with IR. The Chinese term for amniotic fluid (AF) is rooted in the Yin–Yang theory of traditional Chinese medicine, where it symbolizes the inception of human life. Chick early AF (ceAF), a natural product, has shown promise in the field of regenerative medicine. There have been no studies investigating the potential efficacy of ceAF in the treatment of IR-induced damage. This study aims to assess the therapeutic potential of ceAF in alleviating IR-induced damage and elucidate its potential molecular mechanism. **Methods:** In vivo experiments were conducted on 8-week-old male C57BL/6J mice to investigate the effects of ceAF in a radiation injury model induced by whole-body irradiation with X-rays (6 Gy) for 5 min. The ceAF was extracted from chicken embryos aged 7–9 days. **Results:** We found that the supplementation of ceAF reduces mortality induced by IR, improves exercise capacity in IR mice, and reverses IR-induced skin damage. IR leads to varying degrees of volume atrophy and weight loss in the major internal organs of mice. However, ceAF intervention effectively mitigates IR-induced organ damage, with a notable impact on the spleen. The supplementation of ceAF enhances spleen hematopoietic and immune functions by reducing oxidative stress, alleviating inflammatory responses, and preventing splenic DNA damage from IR exposure, ultimately leading to an overall improvement in health. **Conclusions:** ceAF effectively alleviates body damage induced by IR, and our findings provide new perspectives and therapeutic strategies for mitigating IR-induced damage.

## 1. Introduction

Patients exposed to high doses of radiation, whether from cancer treatment or nuclear accidents, may experience acute ionizing radiation (IR) exposure, leading to severe damage to cells, tissues, and organs [1,2]. Exposure to IR can result in systemic damage and increased mortality rates [3]. Although the exact mechanism by which IR causes harm is still unclear, it is believed that reactive oxygen species (ROS) and free radicals, due to radiation exposure, play a significant role [4,5]. The overload of ROS impairs essential cellular components such as proteins, lipids, DNA, and RNA, ultimately leading to abnormal cell apoptosis and necrosis [5]. IR causes extensive, severe, and irreversible damage to internal organs, with the spleen being particularly affected. As the largest peripheral immune organ, the spleen experiences significant oxidative stress and inflammation due to IR exposure, resulting in hematopoietic dysfunction and loss of immune function [6,7]. Protecting the spleen is crucial for minimizing IR-induced damage. However, there are currently no specific drugs available for treating radiation-induced organ harm [8]. While there are nine Food and Drug Administration (FDA)-approved radiation protection agents, including amifostine, that can shield normal tissues from IR-induced damage [9,10], they have limitations such as rapid degradation, vulnerability to acidic conditions, restrictions to intravenous use, and difficulties in protecting the intestines from radiation [10]. Therefore, it is imperative to develop new radioprotective agents to alleviate radiation-induced damage during nuclear accidents or treatments.

Amniotic fluid (AF), a nutrient-rich protective fluid, provides an optimal growth environment for embryos during gestation across various species. The Chinese term for AF is deeply rooted in the Yin–Yang theory of traditional Chinese medicine, symbolizing the inception of human life. Contemporary medical studies have identified AF as primarily composed of water, containing a diverse array of solute components, such as lipids, peptides, amino acids, and nucleic acids, which are crucial for embryonic development and organ formation [11,12,13]. Chick early AF (ceAF) shares similar functions with AF in other species and is easily accessible and cost-effective, making it a valuable resource for investigating the composition and function of AF [14]. Our previous studies have demonstrated that the intravenous injection of ceAF effectively reduces ischemic heart injury in pigs and mice by modulating the Hippo–Yap pathway [15]. Furthermore, it has been shown that ceAF protects the hearts of mice with myocardial infarction by suppressing inflammatory gene expression, decreasing the infiltration of inflammatory neutrophils and macrophages, as well as reducing cardiac apoptosis and fibrosis [16]. Notably, ceAF shows promise in tissue repair following injury and in regenerative medicine [17,18,19]. However, the effect of ceAF on ameliorating IR-induced organ damage, including splenic damage, remains unclear.

The present study aims to investigate the potential of ceAF in enhancing repair mechanisms following IR-induced organ damage. We found that the intraperitoneal injection of ceAF effectively reversed IR-induced organ damage. Furthermore, ceAF facilitated the repair of DNA damage in the spleen via reducing abnormal cell death and inhibiting excessive oxidative stress and inflammatory responses in the spleen. Despite the complexity of ceAF composition and the challenges in identifying specific therapeutic components, the present study suggested that ceAF possesses significant therapeutic properties, low immunogenicity, and ease of administration, rendering it a promising treatment for non-invasive injuries resulting from IR treatment methods.

## 2. Materials and Methods

### 2.1. Animals

Male C57BL/6J mice (age, 8 weeks) were obtained from GemPharmatech Co., Ltd. (Nanjing, China) for the current study. All experimental procedures were conducted according to the principles of the Guide for the Care and Use of Laboratory Animals (National Institutes of Health, 8th edition, 2011) and were approved by the Laboratory Animal Welfare & Ethics Committee of Jiangnan University (approval no. JN.20231007c0500430[474]; Wuxi, China, approval date: 07 October 2023). The mice were housed in a specific pathogen-free laboratory animal room with ad libitum access to commercial rodent chow and water, maintained at a temperature of 20 °C, a relative humidity of 30–60%, and a reversed light/dark cycle of 12:12 h. The mice were euthanized with an intravenous overdose of pentobarbital sodium (200 mg/kg; MilliporeSigma, St. Louis, MO, USA) at the end of the experiment.

### 2.2. Preparation of ceAF

ceAF was isolated from chicken embryos as previously described [15,16]. Briefly, fresh fertilized eggs were washed twice with warm water (37 °C) and maintained at room temperature for 12 h, with the larger end facing upwards. Subsequently, the eggs were transferred to an incubator set at a constant temperature of 37 °C and a humidity level of 80%. The rotation angle of the eggs was adjusted to 45° with a rotation frequency of 4 times/h, and the eggs were continuously incubated for 7–9 days. To extract AF from early chicken embryos under sterile conditions, the larger end of the egg was carefully opened with tweezers, the shell and coat were removed, and the embryos and remaining contents were transferred to a sterile cell culture dish. Using a 1 mL sterile syringe, the amniotic membrane was pierced from the top of the embryo to extract the AF without touching the embryo. Each embryo typically yields 0.8–1.5 mL of AF. Fresh AF was then centrifuged at 1000 rpm for 5 min at 4 °C to eliminate impurities and to obtain AF suitable for experimental purposes. The ceAF was used as soon as possible or was maintained at −80 °C for up to 3 months (Figure S1).

### 2.3. IR-Induced Damage Procedure

The mice were placed in a Rad Source RS2000Prox irradiator (Atlanta, GA, USA) for a 30 min stabilization period before being exposed to 6 whole-body X-ray irradiations for 5 min [20]. Sham-treated control mice underwent the same procedure, with a 30 min stabilization period followed by an additional 5 min without X-ray.

### 2.4. ceAF Intervention and General Index Detection

The mice were randomly assigned to the following four groups: control group (sham + saline), IR group (IR + saline), low-dose ceAF-treated IR group (IR + 4 mL/kg ceAF), or high-dose ceAF-treated IR group (IR + 20 mL/kg ceAF). Intraperitoneal injections of ceAF or normal saline were administered 7 days prior to IR exposure, followed by continuous intraperitoneal injections of normal saline and ceAF for 14 days post-IR. Body weight, daily food intake, mental state, and death of mice were monitored continuously for 14 days after the ionizing irradiation. The number of mouse deaths in each group after IR was recorded to determine the survival rate. The weights of various organs, including the heart, liver, spleen, lungs, kidneys, and thymus, were measured on day 14. Considering that IR causes significant changes in animal body weight, while tibia length remains relatively stable in adult mice, the organ index was calculated as the ratio of organ weight to tibia length.

### 2.5. Exercise Test

The exercise ability of the mice was assessed on days 3, 7, 10, and 14 following the IR treatment. Prior to testing, each mouse was allowed a 10 min acclimation period in the exercise box. Mouse activity was tracked and analyzed using EthoVision^®^XT (Noldus, Wageningen, the Netherlands). The distance traveled, exercise duration, and rest periods were then calculated using the associated MWM analysis software (WMT-100S, v0.6.13).

### 2.6. Hematoxylin and Eosin (H&E) Staining

H&E staining was performed as previously described [21,22]. Briefly, mice were euthanized on day 14 after IR, and the heart, liver, spleen, lungs, and kidneys were subsequently removed. The tissues were fixed with 4% paraformaldehyde, followed by dehydration, paraffin embedding, and sectioning. These sections (10 μm) were then stained with hematoxylin (Solarbio, Beijing, China. #H8070) and eosin (Solarbio, G1100) for histopathological analysis. Images were captured with a light microscope (BX-51, Olympus, Tokyo, Japan).

### 2.7. Analysis of Peripheral Blood

On day 14 post-IR, blood samples were collected from the posterior orbital nerve plexus of the mice using heparin-containing capillaries and transferred to heparin-coated tubes. The blood was thoroughly mixed on a rotating oscillator, and a complete blood count analysis was conducted within 30 min using a fully automated blood analyzer from Mindray Bio-Medical Electronics Co., Ltd. (BC-5000 Vet, Shenzhen, China).

### 2.8. Flow Cytometry Analysis

After 14 days of IR treatment, four mice from each group were euthanized and the spleen was carefully removed. The spleen was filtered through a 40 μm filter using a sterile plunger to create a single-cell suspension. To assess the proportion of CD4^+^ and CD8^+^ T cells, 1 × 10^6^ cells were stained with CD3-APC antibody (1:100, Elabscience, Wuhan, China), CD4 FITC antibody (1:100, Elabscience, Wuhan, China), and CD8 PE antibody (1:100, Elabscience, Wuhan, China) for 30 min in the dark at room temperature. Flow cytometric analysis of the labeled cells was then conducted using a BD FACS AriaIII flow cytometer from BD Biosciences (Franklin Lakes, NJ, USA), and the cells were quantitatively analyzed by FlowJo software (v10.8).

### 2.9. Reverse Transcription–Quantitative Polymerase Chain Reaction (RT-qPCR)

RT-qPCR was conducted as previously described [23]. Total RNA was extracted using TRIzol reagent from Vazyme Biotech Co., Ltd. (Nanjing, China), followed by cDNA synthesis using the HiScript II One Step RT-PCR Kit (Vazyme, Nanjing, China). qPCR was carried out using an SYBR-based method on the LightCycler^®^ 96 System. The expression of the target gene was normalized to *Gapdh* as an internal reference and calculated using the 2^−ΔΔCq^ method. The primers were synthesized by Sangon Biotech Co., Ltd. (Shanghai, China), and their sequences are available in Supplementary Table S1. The RNA quality metrics of this study are provided in Supplementary Table S2.

### 2.10. Western Blot Analysis

Tissue samples were added to RIPA lysis buffer with protease and phosphatase inhibitors from Beyotime Biotechnology (Shanghai, China) and then fully ground in an automatic grinding machine (JXFSTPRP-CLN, Shanghai, China). The supernatant was obtained and protein concentration was determined using the BCA protein assay kit (Thermo Fisher Scientific, Waltham, MA, USA). Total protein samples (30 μg) were then separated by SDS-PAGE on a 12% polyacrylamide gel, transferred to a PVDF membrane, and blocked with 5% skimmed milk for 2 h. The membrane was then incubated with primary antibodies against BCL2, BAX, γH2AX, RAD51, and β-actin from Wanleibio (1:1000; Shenyang, China) overnight at 4 °C, followed by incubation with a horseradish peroxidase-conjugated secondary antibody (1:5000, WanleiBio, Shenyang, China) at room temperature for 1 h. Signal detection was performed using ECL detection reagent (Beyotime, Shanghai, China).

### 2.11. Enzyme Linked Immunosorbent Assay (ELISA)

The IL-1β, IL-6, TNF-α, and 8-oxoguanine (8-oxoG) levels in the serum of the mice were measured using an ELISA kit. Whole blood samples from the mice were incubated at 4 °C for 30 min and then centrifuged at 5000 rpm for 20 min to obtain the supernatant for subsequent experiments. The 8-oxoG levels were determined using a commercial Mouse Mouse 8-oxoguanine (8-OXOG) ELISA Kit from Wuhan Saipei Biotechnology Co., Ltd. (SP14830, Wuhan, China). The IL-1β, IL-6, and TNF-α levels were detected using a commercial Mouse IL-1β (Interleukin 1 Beta) ELISA Kit (M0037), Mouse TNF-α (Tumor Necrosis Factor Alpha) ELISA Kit (M3063), and Mouse IL-6 (Interleukin 6) ELISA Kit (M0044) from Elabscience Biotechnology Co., Ltd. (Wuhan, China).

### 2.12. Statistical Analysis

The experiments were conducted in a randomized and double-blinded manner, and the data are presented as the mean ± SEM. One-way ANOVA, followed by Bonferroni’s post hoc test, was used for multiple comparisons. A *p*-value of less than 0.05 was considered to indicate a statistically significant difference.

## 3. Results

### 3.1. Supplementation with ceAF Reduces IR-Induced Mortality and Enhances the Exercise Capacity of Mice Following IR

**Figure 1:**
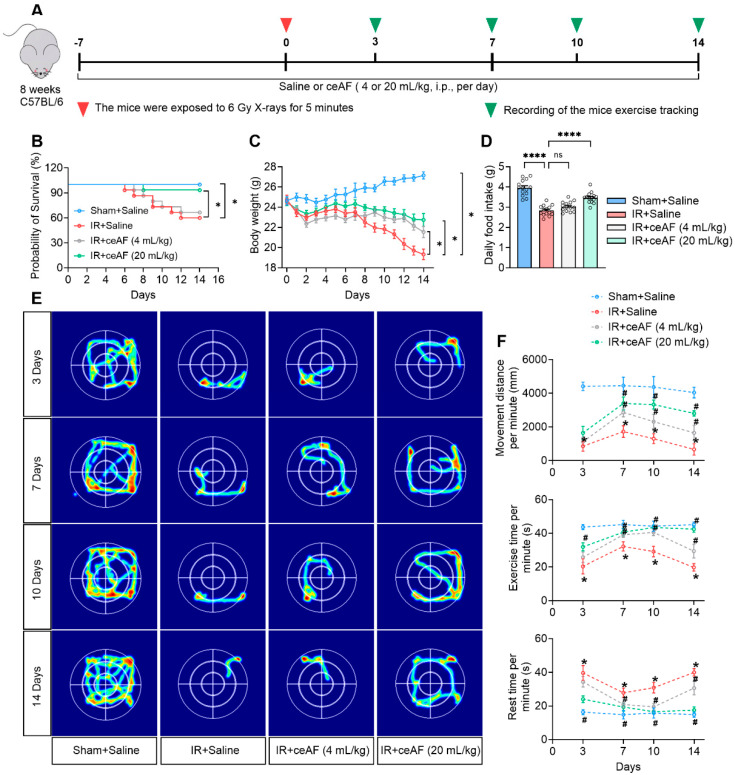
**Supplementation with ceAF reduces IR-induced mortality and enhances the exercise capacity of mice following IR.** (**A**) Schematic diagram of experiments. (**B**–**D**) ceAF effectively promoted the probability of survival (**B**), body weight (**C**), and daily food intake (**D**) in IR-treated mice. (**E**) Representative images of mouse movement track. (**F**) ceAF increased movement distance and exercise time per minute while it decreased resting time in IR-treated mice. n = 5 per group in (**E**,**F**). Data represent the mean ± SEM. * *p* < 0.05; **** *p* < 0.0001. # *p* < 0.05 vs. IR + saline. One-way ANOVA.

To investigate the potential of ceAF supplementation in reducing IR-induced body damage in vivo, saline or two different concentrations of ceAF were administered to sham- or IR-treated mice via intraperitoneal injection. Subsequently, the survival rate, body weight, and daily food intake of the mice were monitored (Figure 1A). It was observed that IR-treated mice administered saline began to die on day 6 post-irradiation, with mortality gradually increasing. Although the mortality rate of IR-treated mice administered low-dose ceAF was higher, it showed a slight improvement compared with the saline-treated group. Notably, the mortality rate of IR-treated mice administered high-dose ceAF was low, reaching levels similar to the sham group (Figure 1B). In addition, the body weight and daily food intake of the mice in the high-dose ceAF-treated group were significantly improved compared with those in the saline-treated IR group (Figure 1C,D).

IR-induced body damage often results in a significant decrease in exercise capacity [24]; therefore, the exercise status of saline- and ceAF-treated mice was continuously assessed. The results indicated that IR led to a notable deterioration in the motor function of mice, as evidenced by a decrease in movement distance and exercise time per minute, along with an increase in rest time (Figure 1E). Notably, both doses of ceAF intervention effectively ameliorated the motor deficits in IR-treated mice, with the high dose showing a more pronounced effect (Figure 1F). Taken together, these results suggested that ceAF has promising potential in improving IR-induced body damage.

### 3.2. Supplementation with ceAF Reverses IR-Induced Skin Damage in Mice

**Figure 2:**
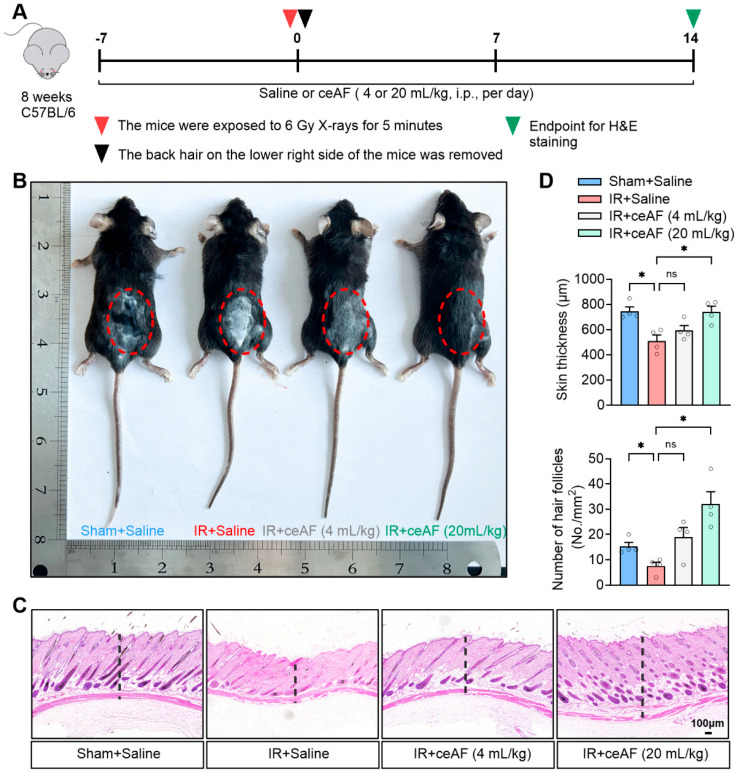
**Supplementation with ceAF reverses IR-induced skin damage in mice.** (**A**) Schematic diagram of experiments. (**B**) Representative images of mouse hair growth. (**C**) Representative images of H&E staining of skin tissues. (**D**) ceAF enhanced skin thickness and increased the number of hair follicles in IR-treated mice. n = 4 per group. Data represent the mean ± SEM. * *p* < 0.05. One-way ANOVA.

Skin, as the most superficial organ of the human body, is highly sensitive to IR. Notably, 95% of patients undergoing tumor radiotherapy in clinical settings experience some degree of skin damage; this damage is a direct result of IR exposure and serves as a key indicator of the extent of radiation-induced harm [25]. The present study demonstrated that IR led to inhibited hair growth or hair growth arrest in mice, whereas intervention with ceAF promoted hair growth in IR-exposed mice. Notably, high-dose ceAF treatment completely reversed the hair growth arrest caused by IR (Figure 2B). The histological examination of skin tissue through H&E staining revealed that skin tissue in normal mice exhibited a healthy appearance, with skin appendages such as hair follicles and sweat glands evenly distributed. IR treatment, on the other hand, led to a decrease in skin thickness in mice and a significant reduction in hair follicles and other skin appendages, potentially explaining the observed stagnation of hair growth. ceAF intervention effectively restored the structure of skin tissue and increased the number of hair follicles, with high-dose ceAF treatment yielding even more significant results (Figure 2C,D). These findings indicated that ceAF may effectively mitigate the direct damage caused by IR exposure to the body.

### 3.3. ceAF Ameliorates IR-Induced Organ Injury

**Figure 3:**
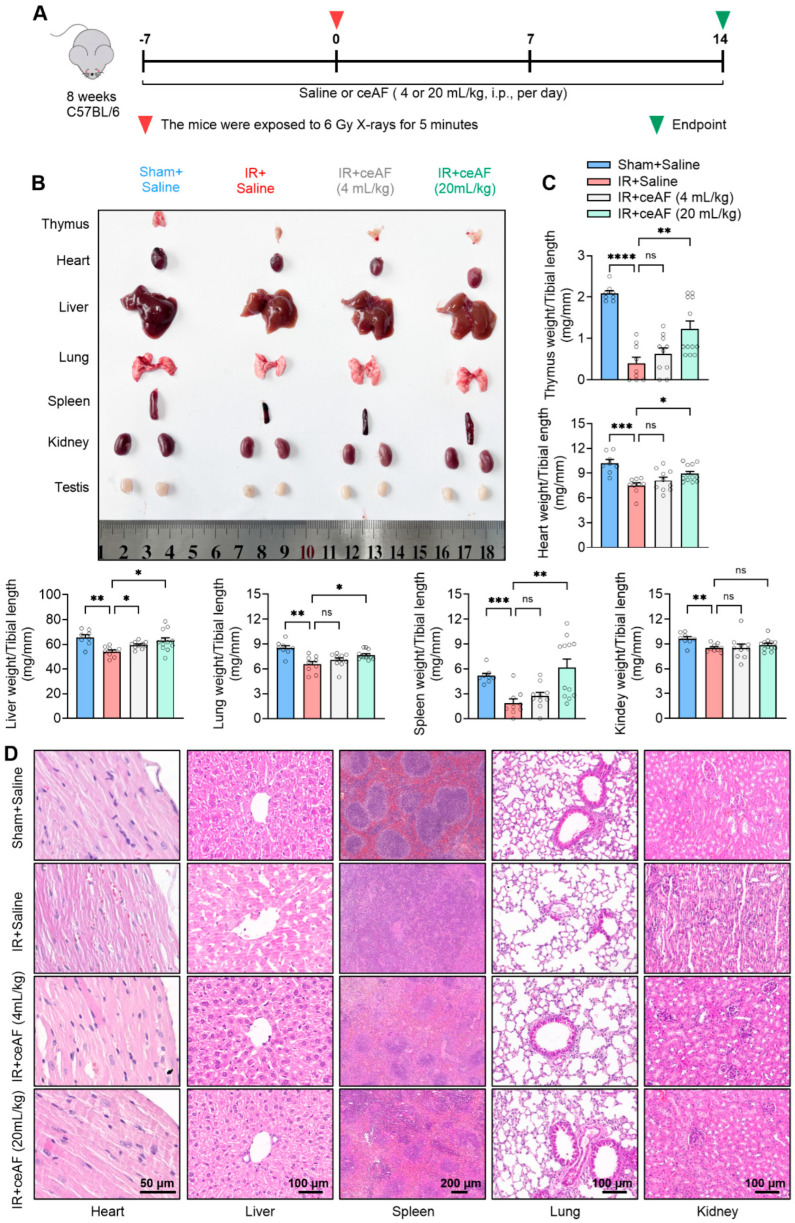
**ceAF ameliorates IR-induced organ injury.** (**A**) Schematic diagram of experiments. (**B**) Representative images of mouse organ, including thymus, heart, liver, lung, spleen, kidney, and testis. (**C**) ceAF increased organ weight in IR-treated mice. (**D**) Representative images of H&E staining of organ tissues. n = 8, 9, 10, 12 per group in (**C**). n = 4 per group in (**D**). Data represent the mean ± SEM. * *p* < 0.05; ** *p* < 0.01; *** *p* < 0.001; **** *p* < 0.0001. One-way ANOVA.

IR-induced damage often manifests systemic characteristics, particularly affecting the internal organs of the body to a significant degree. The mortality rate of mice following 2 weeks of IR treatment was close to 50%. Subsequently, the experiment was terminated after a 2-week administration of IR-treated mice with saline or ceAF, and the damage to the major internal organs of the mice was assessed (Figure 3A). The present findings indicated that IR could induce varying degrees of volume atrophy and weight loss in major internal organs, such as the thymus, heart, liver, spleen, lungs, kidneys, and testicles, in mice. The morphological observations revealed that most of the organs of the IR-treated mice administered saline appeared pale, suggesting insufficient blood infiltration, possibly linked to severe spleen atrophy (Figure 3B). Following ceAF intervention, the reduced internal organ volume and weight in IR-treated mice were effectively reversed, with a more pronounced therapeutic effect observed in the high-dose group (Figure 3B,C). Further organ pathology tests demonstrated that IR caused disordered myocardial arrangement, liver cell swelling and fission, severe structural damage to the red and white pulp of the spleen, reduced alveolar breakage, pulmonary vascular remodeling, and nephron damage in mice, all of which were mitigated by ceAF treatment, especially in the high-dose ceAF group (Figure 3D). These results indicated that ceAF may effectively mitigate the internal organ damage induced by IR.

### 3.4. ceAF Relieves IR-Induced Injury of the Spleen Hematopoietic System

**Figure 4:**
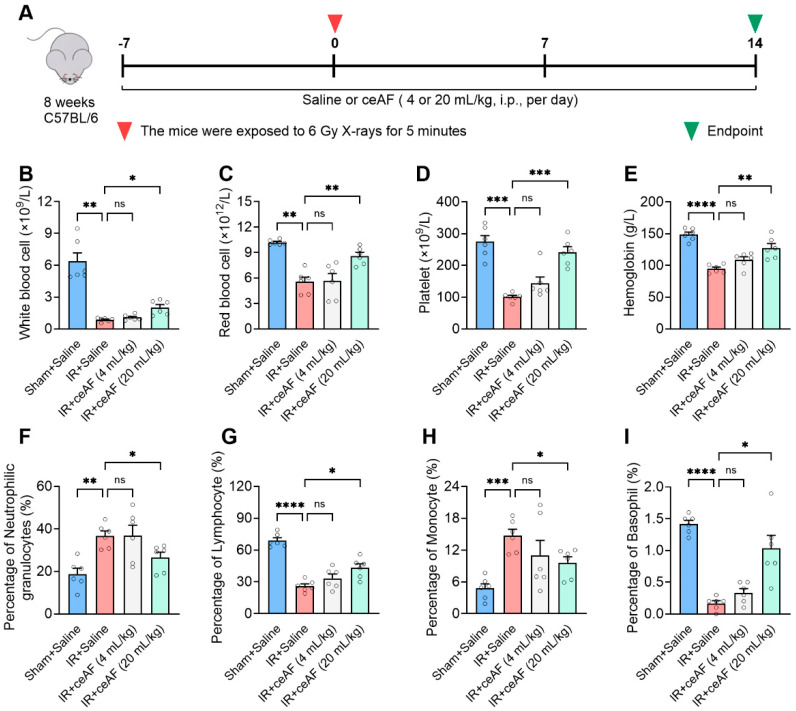
**ceAF relieves IR-induced injury of the spleen hematopoietic system.** (**A**) Schematic diagram of experiments. ceAF increased the major blood cell types, such as white blood cells (**B**), red blood cells (**C**), platelets (**D**), hemoglobin (**E**), lymphocytes (**G**), and basophil (**I**). Additionally, ceAF decreased neutrophils (**F**) and monocytes (**H**) in IR-treated mice. n = 6 per group. Data represent the mean ± SEM. * *p* < 0.05; ** *p* < 0.01; *** *p* < 0.001; **** *p* < 0.0001. One-way ANOVA.

The impact of IR on the hematopoietic system is profound and direct. Previous observations have shown that IR-induced mice exhibit pallor in almost all internal organs, with significant damage to the red pulp of the spleen. Since hematopoietic function is vital for overall health, the present study further investigated the effects of ceAF on the composition and proportion of blood cells (Figure 4A). The experimental findings demonstrated that ceAF intervention could successfully reverse the decline in major blood cell types, including white blood cells, red blood cells, platelets, and hemoglobin induced by IR. Additionally, the abnormal elevation in neutrophils and monocytes triggered by IR was effectively suppressed by ceAF (Figure 4B–I). These results indicated that ceAF may exert a substantial protective influence on spleen hematopoiesis.

### 3.5. ceAF Improves Spleen Immune Function in IR-Treated Mice

**Figure 5:**
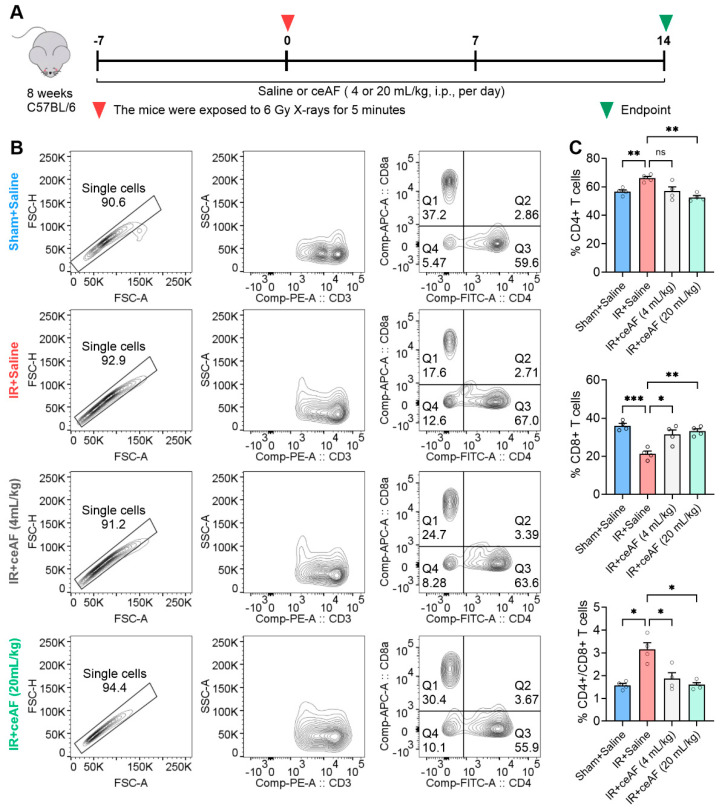
**ceAF improves spleen immune function in IR-treated mice.** (**A**) Schematic diagram of experiments. (**B**,**C**) ceAF effectively reduced CD4^+^ T cells, increased CD8^+^ T cells, and normalized the ratio of CD4^+^/CD8^+^ T cell of spleen in IR-treated mice. n = 6 per group. Data represent the mean ± SEM. * *p* < 0.05; ** *p* < 0.01; *** *p* < 0.001. One-way ANOVA.

The spleen, being the largest peripheral immune organ, serves a critical role in the immune response [26]. Previous observations have demonstrated that IR leads to significant damage to the white pulp of the spleen, which is the primary site for specific immunity. These findings prompted further investigation into whether ceAF has a regulatory effect on immune function mediated by spleen lymphocytes in IR-treated mice. It has previously been indicated that the ratio of spleen CD4^+^/CD8^+^ T cells serves as a key indicator of immune regulation [27]. The present study used flow cytometry to analyze CD4^+^ T cells and CD8^+^ T cells and their ratio in the spleens of IR-treated mice administered either saline or ceAF (Figure 5A). The experimental findings revealed that IR resulted in an increase in CD4^+^ T cells, a decrease in CD8^+^ T cells, and a significant rise in the CD4^+^/CD8^+^ T-cell ratio in mice, indicating the severe impairment of spleen immune function. Notably, intervention with ceAF effectively reduced CD4^+^ T cells, increased CD8^+^ T cells, and normalized the CD4^+^/CD8^+^ T-cell ratio (Figure 5B,C). These results suggested that ceAF not only enhances the hematopoietic function of the spleen in IR-treated mice but also efficiently restores immune function.

### 3.6. ceAF Suppresses IR-Induced Oxidative Stress and Inflammation and Effectively Enhances DNA Repair After IR-Induced Damage to the Spleen

**Figure 6:**
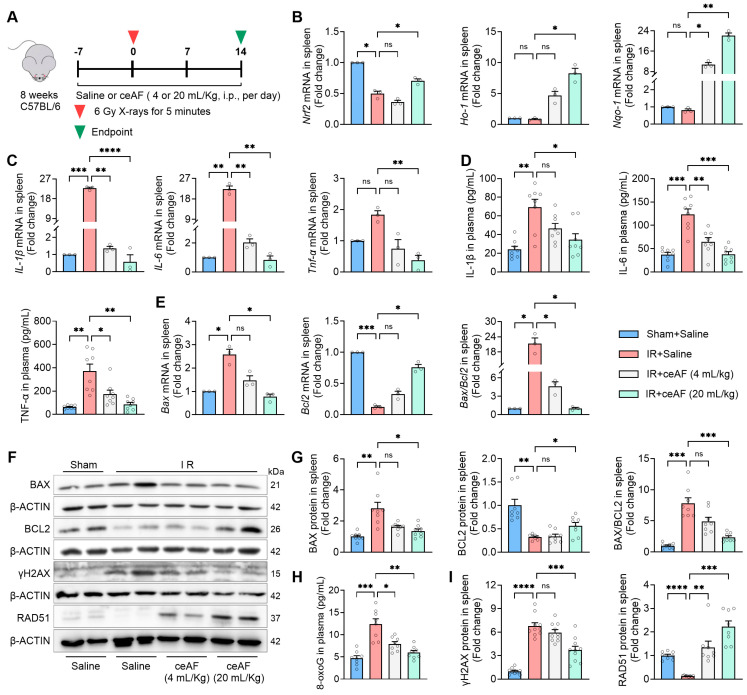
ceAF suppresses IR-induced oxidative stress and inflammation and enhances DNA repair after IR-induced damage to the spleen. (**A**) Schematic diagram of experiments. (**B**) ceAF upregulated the expression of antioxidant genes *Nrf2*, *Ho-1*, and *Nqo-1* in IR-treated mouse spleen. (**C**) ceAF downregulated the expression of pro-inflammatory factors *Il-1β*, *Il-6*, and *Tnf-α* in IR-treated mouse spleen. (**D**) ceAF decreased the expression of pro-inflammatory factors IL-1β, IL-6, and TNF-α in IR-treated mouse plasma. (**E**) ceAF inhibited the expression of *Bax* while it promoted the expression of *Bcl2* in IR-treated mouse spleen. (**F**) The expression of BAX, BCL2, γH2AX, and RAD51 in spleen detected by Western blot. (**G**) Statistical analysis of the expression of BAX and BCL2 from Western blot. (**H**) ceAF decreased the levels of 8-oxoG in IR-treated mouse plasma. (**I**) Statistical analysis of the expression of γH2AX and RAD51 from Western blot. n = 3 per group in (**B**,**C**,**E**). n = 8 per group in (**D**,**G**). n = 10 per group for detecting γH2AX protein. n = 8 per group for examining BAX, BCL2, and RAD51 protein. Data represent the mean ± SEM. * *p* < 0.05; ** *p* < 0.01; *** *p* < 0.001; **** *p* < 0.0001. One-way ANOVA.

Considering the significant protective effects of ceAF on the spleen in IR-treated mice, the present study investigated the molecular effects of ceAF on IR-induced spleen injury. Previous studies have indicated that IR leads to an increase in ROS, which in turn triggers inflammation and exacerbates overall damage [28,29]. Therefore, the current study focused on the impact of ceAF on IR-induced oxidative stress and inflammation in the spleen (Figure 6A). The results showed that IR decreased the expression levels of antioxidant genes, including nuclear factor-erythroid 2-related factor 2 (*Nrf2*), heme oxygenase 1 (*Ho-1*), and NAD(P)H quinone oxidoreductase 1 (*Nqo-1*), while simultaneously increasing the expression levels of pro-inflammatory factors, such as interleukin-1β, interleukin-6, and tumor necrosis factor-α. Notably, ceAF effectively reversed these effects, particularly at a higher dose (Figure 6B–D). Additionally, IR can directly damage DNA and increase cell apoptosis. The present study assessed the role of ceAF in DNA damage, repair, and apoptosis in the spleen. IR elevated the DNA damage marker protein γH2AX and pro-apoptosis marker protein BAX while reducing the repair marker protein RAD51 and anti-apoptotic factor BCL2. Notably, ceAF treatment mitigated IR-induced DNA damage and apoptosis, enhancing post-damage repair (Figure 6E–H). These findings indicated that ceAF may decrease IR-induced body damage by reducing oxidative stress, inflammation, and DNA damage in the spleen.

## 4. Discussion

Medical radioactive sources and increasing nuclear contamination result in individuals being at a higher risk of radioactive injury [30]. IR often leads to extensive and severe damage to the body, and while the exact mechanism of IR-induced damage is not fully understood, the excessive production of ROS and direct DNA damage are considered major contributors [4,5]. IR poses a significant threat to DNA integrity. On the one hand, high-energy particles from IR can directly damage DNA by breaking phosphodiester bonds or glycosidic bonds. On the other hand, IR ionizes water molecules within the cell, generating a multitude of reactive free radicals that interact with DNA bases, leading to oxidative damage and the formation of 8-oxoG. Furthermore, the lack of an effective clinical drug to treat IR-induced injury poses a challenge [31]. Amifostine, the earliest FDA-approved radiation protection drug for clinical use, has limited effectiveness and is susceptible to metabolic failure [9,10]. Therefore, there is an urgent need to discover improved products for the prevention and treatment of IR-induced injury. The present study highlighted the significant therapeutic potential of ceAF, a natural product, in the management of IR-induced injury. The findings indicated that ceAF could enhance the hematopoietic and immune functions of the spleen by reducing oxidative stress, alleviating inflammatory responses and preventing DNA damage caused by IR exposure, ultimately leading to an overall improvement in health. With its low immunogenicity, ease of administration, and notable therapeutic benefits, ceAF has emerged as a promising treatment option for non-invasive injuries resulting from IR exposure.

Our previous studies have demonstrated that ceAF effectively reduces ischemic heart damage in pigs and mice and shows significant protective effects on mice with acute myocardial infarction, which is closely related to ceAF, inhibiting inflammation and reducing myocardial fibrosis [15,16]. In addition, recent research has indicated that ceAF serves a vital role in wound healing by accelerating cell proliferation and migration. Furthermore, structural analogs of guanosine and deoxyinosine derived from ceAF have been identified as key substances that promote skin wound healing [17]. Additionally, ceAF has been shown to decrease inflammation and oxidative stress through the TLR4/NF-κB and Nrf2 pathways, enhancing diabetic wound healing [19]. These findings collectively highlight the potential of ceAF in regenerative medicine. The present study established a link between ceAF and IR-induced damage repair and revealed that ceAF exerts a beneficial impact on reversing damage caused by IR. This underscores the efficacy of ceAF in tissue and body regeneration, offering promising implications for the development of anti-injury drugs utilizing ceAF. However, similar to prior studies, the present study did not identify the specific substances within ceAF responsible for its anti-IR-induced damage effects, likely due to the complex composition of ceAF. Nonetheless, it could be hypothesized that the anti-damage properties of ceAF may involve multiple substances rather than a single or several components, warranting further investigation. In addition, to reduce the complexities that may arise from gender differences (such as hormonal fluctuations) and age-related variables (such as immune system maturity) in preliminary exploratory research, we opted for ceAF to focus on a single gender and age group. However, gender and age differences may have a potential impact on this study’s results. In our future research, we will systematically include female mice, different age groups, and gender comparison experiments to comprehensively assess the regulatory effects of these factors on ceAF in response to IR.

Notably, ceAF treatment significantly increased the weight of the spleen and thymus. It could be speculated that the significant increase in the weight of the spleen and thymus caused by ceAF, in contrast with the relatively small increase in the weight of other organs, may be attributed to two primary reasons. Firstly, numerous studies have demonstrated that the immune system is highly sensitive to IR [1,32]. Central immune organs, such as the thymus [33,34], and peripheral immune organs, such as the spleen [35,36], experience rapid damage when exposed to acute and severe IR, often resulting in swift and progressive atrophy. In the present study, it was observed that the thymus and spleen of some IR-treated mice exhibited significant atrophy or even completely disappeared. By contrast, solid organs, such as the heart, lungs, and kidneys, are more resilient and do not exhibit severe atrophy. ceAF treatment-induced increases in the mass of the thymus and spleen may be due to the fact that the mass loss of these two organs was more obvious following IR. Secondly, it was noted that ceAF-administered IR treatment significantly enhanced the mass of the thymus and spleen, with some ceAF-treated IR mice displaying compensatory enlargement of the spleen. These findings suggested that ceAF may have a substantial protective effect on the immune system. In light of these observations, we are investigating the regulatory effects of ceAF on immune rejection following lung and other internal organ transplants and have obtained promising data.

A pertinent question to consider is the determination of the optimal dose of ceAF for the treatment of IR-induced damage. In the present study, two different doses, 4 and 20 mL/kg/day, were administered intraperitoneally to IR-treated mice. Both low-dose and high-dose ceAF exhibited protective effects against IR-induced damage; this is evidenced by the enhancement of survival rates and exercise capacity in IR-treated mice, as well as improvements in the hematopoietic and immune functions of the spleen. Additionally, ceAF reduced oxidative stress and inflammatory responses caused by IR while also preventing DNA damage in the spleen resulting from IR exposure. Ultimately, these effects contribute to an overall improvement in health. However, the effects observed in the high-dose group (20 mL/kg/day) were more pronounced. Our laboratory has previously administered doses of ceAF at 1.5, 5.0, and 15.0 mL/kg to treat cardiac ischemia–reperfusion injury in mice; it was revealed that the 5.0 mL/kg dose exhibited notable protective effects, with even more significant results observed in response to the 15.0 mL/kg dose [15,16]. Moreover, several studies have indicated that a 5 or 10% dose of ceAF can effectively promote wound healing resulting from various factors and mechanisms [17,19]. While it is not possible to identify the ideal treatment dose due to factors such as the severity of the patient’s condition, previous research on ceAF, along with the present experimental data, suggests that a dose range of 10–20 mL/kg ceAF may represent an optimal therapeutic dose. A limitation of this study is the lack of research on the toxicity of ceAF, despite studies indicating its characteristics of being sterile, non-toxic, and having low immunogenicity. We will include this important result in future studies.

Sourced from various avian and mammalian embryos, AF plays a crucial role in protecting fetal development. Research has indicated that human-derived AF or stem cells of AF have a notable impact on damage repair and wound healing [11,37,38,39]. One composition study revealed that the AFs from chicken, duck, and turkey embryos of similar ages all contain high concentrations of IGF-I/II, a key growth factor [40]. These findings suggested that early embryonic AFs from different sources may also have beneficial effects against IR-induced injury, which requires further investigation. However, the specific components of ceAF that reverse IR-induced damage have not yet been elucidated. Studies have reported that the fibroblast growth factor (FGF) family, including FGF2 [41], FGF7, FGF10 [42], and FGF21 [43], all have protective effects on IR-induced damage. In addition, the epidermal growth factor family [44,45] and platelet-derived growth factor [46,47] have also been reported to be related to repair after IR-induced injury. We aim to further explore whether the combination of ceAF and these substances may achieve a more significant therapeutic effect on IR-induced injury. Moreover, studies have demonstrated the therapeutic potential of AF in various post-injury repair scenarios through different administration routes, such as intravenous injection, wound application, and oral administration. The present study specifically highlighted the reversal of IR-induced damage with the intraperitoneal injection of ceAF. By considering these diverse administration methods, it is evident that ceAF has the potential to be developed into a non-invasive treatment. Another important aspect of the present study is the promising results of pre-treating mice with ceAF, demonstrating its potential in preventing IR-induced damage.

The present study not only observed the positive effect of ceAF on hair regeneration and survival rate in IR-induced mice but also highlighted its protective effects on the comprehensive IR-induced damage of internal organs, particularly focusing on the reparative function of the spleen. Morphological analysis revealed that ceAF almost completely reversed spleen atrophy and structural damage induced by IR. Functional testing further demonstrated that ceAF significantly enhanced the hematopoietic and immune functions of the spleen, indicating a potential positive effect on overall immune function. Molecular experiments further elucidated that ceAF mitigated oxidative stress, inflammatory responses, DNA damage, and abnormal cell apoptosis in the spleens of IR-treated mice, thus leading to an overall health improvement. These findings collectively suggest a promising role for ceAF in targeting spleen function and enhancing the repair capacity in the body. Finally, we acknowledge that while this study provides valuable insights into the potential therapeutic role of ceAF in IR-induced damage, further validation of our findings through rigorous experimentation using diverse animal models and human-relevant systems is essential before considering its clinical implications.

**Figure 7:**
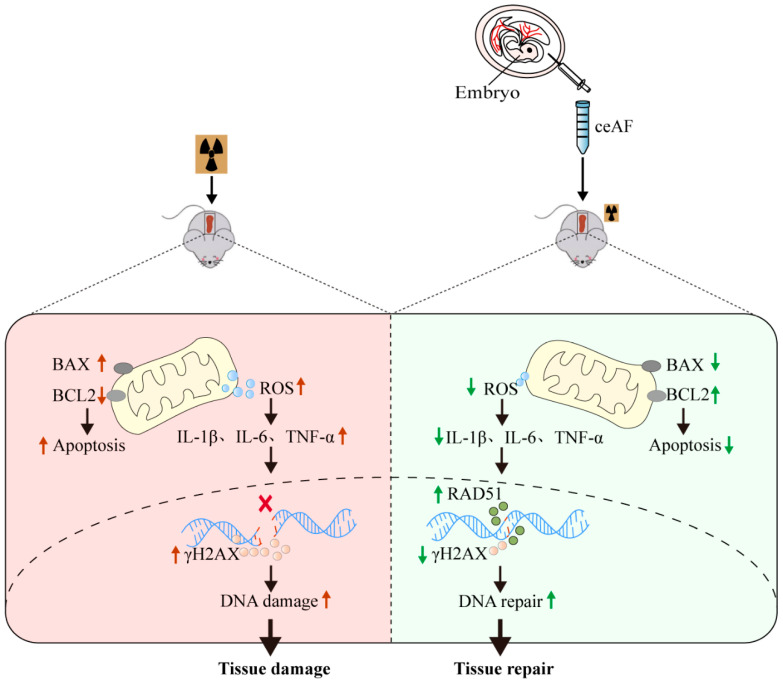
ceAF effectively alleviates IR-induced damage by reducing the level of ROS and pro-inflammatory factor and subsequently enhances the ability of DNA repair after damage in the spleen.

The present study demonstrates that ceAF can effectively alleviate body damage induced by IR. This is evidenced by improvements in exercise capacity, reduced mortality rates, promotion of hair regeneration, restoration of internal organ structure and function, particularly the spleen, as well as decreased oxidative stress, inflammation, and DNA damage in the spleen (Figure 7). These findings offer novel insights and treatment approaches for mitigating IR-induced damage and are particularly relevant in addressing the growing concern of radioactive damage resulting from nuclear contamination.

## Data Availability

All data generated or analyzed during this study are included in this published article.

## Supplementary Materials

The following supporting information can be downloaded at: https://www.mdpi.com/article/10.3390/biomedicines13051253/s1, Figure S1: The protocol for extraction of chick early amniotic fluid (ceAF); Table S1: Primers for qRT-PCR analysis in spleen tissue of mice; Table S2: The RNA quality metrics of spleen.
