# Supplementary material for: Therapeutic Potential of Chick Early Amniotic Fluid in Mitigating Ionizing-Radiation-Induced Damage
Source: Biomedicines. 2025 May 21;13(5):1253. doi: 10.3390/biomedicines13051253 (PMC12109126; doi:10.3390/biomedicines13051253)
Supplement: Supplementary file 1 [file biomedicines-13-01253-s001.zip › biomedicines-3622814-supplementary.pdf]

Supplemental Material

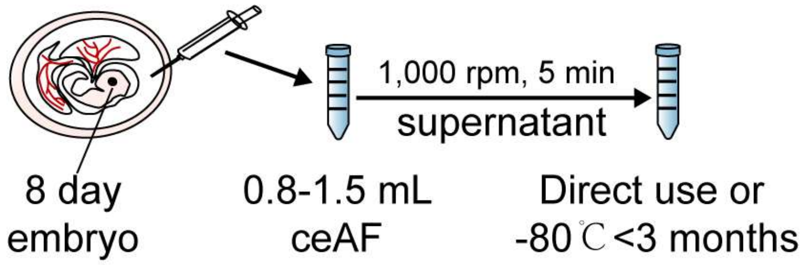

Figure S1. The protocol for extraction of chick early amniotic fluid (ceAF). The larger end of the egg was carefully opened with tweezers, the shell and coat were removed, and the embryos and remaining contents were transferred to a sterile cell culture dish. Using a 1-mL sterile syringe, the amniotic membrane was pierced from the top of the embryo to extract the AF without touching the embryo. Each embryo typically yields 0.8-1.5 mL of AF. Fresh AF was then centrifuged at 1,000 rpm for 5 min at 4 ˚C to eliminate impurities and to obtain AF suitable for experimental purposes. The ceAF was used as soon as possible, or was maintained at -80 ˚C for up to 3 months.

Supplemental Table

Table S1. Primers for qRT-PCR analysis in spleen tissue of mice.

Gene	Species	Primer	Sequence
Nrf2	Mouse	Forward	CTTTAGTCAGCGACAGAAGGAC
		Reverse	AGGCATCTTGTTTGGGAATGTG
Ho-1	Mouse	Forward	GCTAGCCTGGTGCAAGATACT
		Reverse	TGGGGGCCAGTATTGCATTT
Nqo-1	Mouse	Forward	AGGATGGGAGGTACTCGAATC
		Reverse	TGCTAGAGATGACTCGGAAGG
Il-1β	Mouse	Forward	GAAATGCCACCTTTTGACAGTG
		Reverse	TGGATGCTCTCATCAGGACAG
Il-6	Mouse	Forward	TAGTCCTTCCTACCCCAATTTCC
		Reverse	TTGGTCCTTAGCCACTCCTTC
Tnf-α	Mouse	Forward	GACGTGGAACTGGCAGAAGAG
		Reverse	TTGGTGGTTTGTGAGTGTGAG
Bax	Mouse	Forward	TGAAGACAGGGGCCTTTTTG
		Reverse	AATTCGCCGGAGACACTCG
Bcl2	Mouse	Forward	GTCGCTACCGTCGTGACTTC
		Reverse	CAGACATGCACCTACCCAGC
Gapdh	Mouse	Forward	AGGTCGGTGTGAACGGATTTG
		Reverse	TGTAGACCATGTAGTTGAGGTCA

Table S2. The RNA quality metrics of spleen.

Group	Sample	Volume (μL)	Concentration (ng/μL)	OD260/280
Sham+Saline	1	50	698.36	2.02
	2	50	724.15	1.98
	3	50	674.85	1.87
IR+Saline	1	50	700.21	1.95
	2	50	689.12	2.00
	3	50	732.52	1.88
IR+ceAF (4 mL/kg)	1	50	665.34	1.92
	2	50	643.89	1.95
	3	50	687.55	2.00
IR+ceAF (20 mL/kg)	1	50	671.96	1.79
	2	50	692.10	1.84
	3	50	689.81	1.96
